# Suspected Chromium-Induced Acute Pancreatitis: Highlighting the Risks of Over-the-Counter Supplements

**DOI:** 10.14309/crj.0000000000002079

**Published:** 2026-04-16

**Authors:** Yusuf Kagzi, Preethi L. Chintagunta, Saurabh K. Bansal, Imran L. Balouch

**Affiliations:** 1 Department of Internal Medicine, University of Illinois College of Medicine, Peoria, IL; 2 Department of Gastroenterology, University of Illinois College of Medicine, Peoria, IL

**Keywords:** acute pancreatitis, drug-Induced pancreatitis, supplement-induced pancreatitis, chromium polynicotinate, nutraceuticals

## Abstract

Drug-induced pancreatitis accounts for a small percentage of acute pancreatitis cases, and supplement-induced cases are poorly characterized. We report a 59-year-old woman presenting with acute interstitial pancreatitis after recent initiation of an over-the-counter supplement containing chromium polynicotinate and herbal additives. She lacked traditional risk factors, and imaging showed no biliary disease. Lipase exceeded 3000 U/L, and serum chromium was elevated (0.5 ng/mL). Symptoms improved with supportive care and supplement discontinuation. This case suggests a potential association between chromium-containing supplements and pancreatitis, highlighting the need for thorough supplement histories in idiopathic cases and greater awareness of possible pancreatic toxicity from nutraceuticals.

## INTRODUCTION

Acute pancreatitis (AP) is a common gastrointestinal emergency with well-established etiologies; however, up to 20% of cases remain idiopathic. This has prompted investigation into less common causes, including drug- and supplement-induced pancreatitis (DIP; SIP), for which data remain limited. Chromium, a trace mineral found in many over-the-counter supplements, has been shown to accumulate in the pancreas and induce inflammation, though human data are sparse.^1,2^ We report a case of acute interstitial pancreatitis temporally associated with chromium-containing supplementation.

## CASE REPORT

A 59-year-old woman with gastroesophageal reflux disease presented with 4 hours of severe epigastric pain radiating to the back, accompanied by nausea and vomiting. She denied any diaphoresis, tremors, confusion, diarrhea, or presyncope. She also denied alcohol use, smoking, prior pancreatitis, trauma, malignancy, autoimmune disease, or infection. She began taking an over-the-counter “blood sugar support” supplement 3 days before symptom onset. The formulation contained chromium polynicotinate 500 mcg per serving (1,429% of the recommended daily value), along with basil extract, fenugreek, and ginseng. She reported taking once daily, with no dose change or prior exposure to chromium supplementation. Her only prescribed medication was a transdermal estradiol patch and was not taking any other glucose-lowering medications.

**Figure 1.**
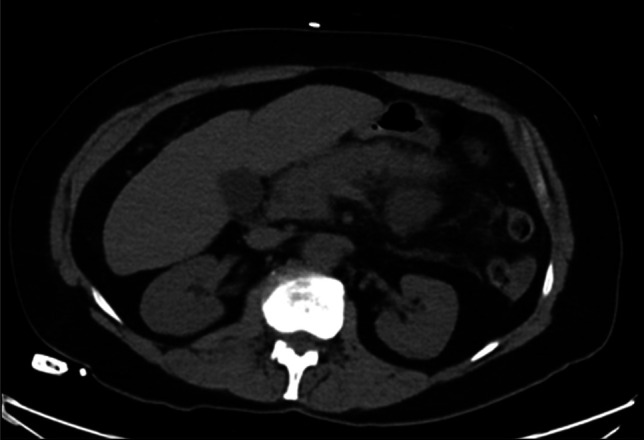
Image showing thickened pancreatic parenchyma with diffuse peripancreatic fat stranding and edema.

On examination, she was afebrile and hemodynamically stable with epigastric tenderness. Laboratory studies showed leukocytosis (13,000/μL) and lipase >3000 U/L, with normal triglycerides, autoimmune panel, and liver enzymes. Serum glucose was 191 mg/dL with a glycated hemoglobin of 5.3%, consistent with stress hyperglycemia. A right upper quadrant ultrasound was negative for gallstones or biliary sludge. There was no gallbladder wall thickening, and the common bile duct measured <6 mm in diameter without dilation. Contrast-enhanced computed tomography (CT) on hospital day 1 revealed acute interstitial edematous pancreatitis without biliary pathology (Figure 1).

**Figure 2.**
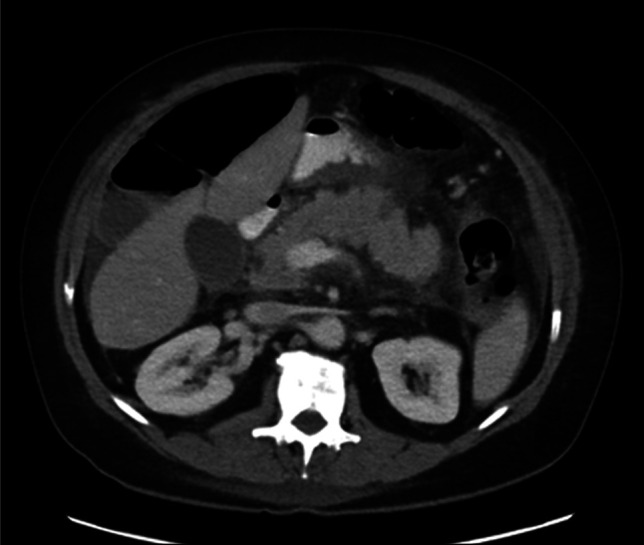
Image showing interstitial edematous pancreatitis with diffuse peripancreatic fat stranding and edema with small volume ascites.

**Figure 3.**
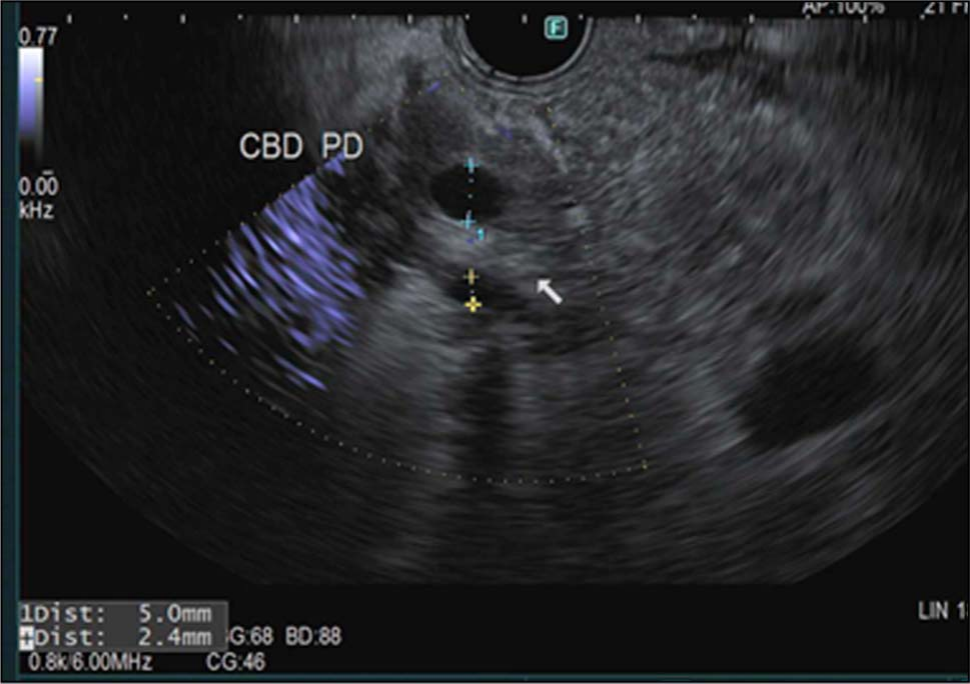
Endoscopic ultrasound image demonstrating a normal common bile duct measuring 5 mm. CBD, common bile duct; PD, pancreatic duct.

She was treated supportively with intravenous fluids, antiemetics, and opioid analgesia. Repeat CT due to persistent abdominal pain on day 4 showed persistent interstitial pancreatitis with mild ascites and reactive gastric antral thickening (Figure 2). A serum chromium level was elevated at 0.5 ng/mL (reference <0.3 ng/mL). Symptoms improved by day 5, allowing advancement to a low-fat diet. The supplement was discontinued, and she was discharged on day 6. At 14-day follow-up, she remained clinically improved. Three months after she underwent secretin-enhanced magnetic resonance cholangiopancreatography (MRCP) and endoscopic ultrasound. MRCP showed improving inflammatory changes without pancreatic duct obstruction. The main pancreatic duct measured 3 mm at baseline with appropriate dilation to 7 mm following secretin administration, consistent with preserved exocrine function. No biliary ductal dilation or choledocholithiasis was identified. Endoscopic ultrasound revealed mild parenchymal changes; pancreatic duct was normal; the common bile duct measured 5 mm without stones, sludge, or obstruction (Figure 3). Given exclusion of alternative etiologies and temporal supplement exposure, possible chromium-associated SIP was suspected.

## DISCUSSION

Although DIP is increasingly recognized, SIP remains underreported, particularly with over-the-counter nutraceuticals. In our case, imaging and laboratory evaluation excluded common etiologies, including biliary disease, malignancy, hypertriglyceridemia, infection, and autoimmune causes. Estrogen therapy is a rare cause of pancreatitis, typically mediated by hypertriglyceridemia; however, this patient had normal lipid levels and was using a transdermal formulation, which is less likely to affect triglyceride metabolism.^2–5^

While over 500 medications have been linked to DIP, herbal and nutritional supplements remain underrepresented in the literature. Chromium, in particular, has not been well documented as a causative agent. This case suggests a potential association between acute interstitial pancreatitis and chromium-containing supplementation in the absence of traditional risk factors, highlighting the need for increased clinical vigilance and patient education.^2,6^

Chromium, a trace mineral, is widely used for metabolic support and is generally considered safe at nutritional doses; however, safety at supraphysiologic doses or in specific formulations remains unclear. The patient's intake of 500 mcg daily (1,429% daily value) and elevated serum chromium level support recent exposure and a possible contributory role.^6–8^

The diagnosis of DIP or supplement-induced pancreatitis remains clinical, relying on temporal association, exclusion of alternative etiologies, and improvement after withdrawal of the suspected agent. Causality assessment tools such as the Naranjo adverse drug reaction probability scale and the Badalov classification provide structured frameworks. The Naranjo algorithm applies a 10-question scoring system tailored to pancreatitis, allowing for objective classification of causality. Scores <2 suggest doubtful DIP, 3–5 indicate possible DIP, 6–8 probable, and >9 highly probable. In our case, the total score is approximately 7 out of 10, placing this case in the probable category for drug-induced pancreatitis per the modified Naranjo scale.^9^ Naranjo Scale and their assigned scores are presented in Table 1. Similarly, Badalov classification originally developed for medication-induced pancreatitis was applied conceptually to supplement exposure. It ranges from Class Ia (strongest evidence: rechallenge and other causes excluded) to Class IV (weakest: only 1 report, no rechallenge or latency). Our case fits into Badalov Class Ib or II, given the exclusion of other causes, temporal relationship, and plausible biological mechanism.^10^ Although rechallenge was not performed, the case fulfills several criteria supporting a probable association and their applicability to this case are summarized in Table 2.

**Table 1.** Naranjo adverse drug reaction probability scale applied to this case

Naranjo question	Response	Points
1. Are there previous conclusive reports on this reaction?	No	0
2. Did the adverse event appear after the suspected agent was administered?	Yes	+2
3. Did the adverse reaction improve when the agent was discontinued?	Yes	+1
4. Did the adverse reaction reappear upon readministration?	Not performed	0
5. Are there alternative causes that could have caused the reaction?	No reasonable alternative identified	+2
6. Did the reaction reappear with placebo?	Not applicable	0
7. Was the agent detected in blood at toxic concentrations?	Serum chromium elevated above reference range	+1
8. Was the reaction more severe with increased dose or less severe with decreased dose?	Not assessable	0
9. Did the patient have a similar reaction to the same or similar agents previously?	No	0
10. Was the adverse event confirmed by objective evidence?	Imaging-confirmed acute pancreatitis	+1
Total score		7 (probable adverse drug reaction)

**Table 2.** Application of the Badalov classification to chromium supplement exposure

Badalov criterion	Present in this case	Supporting evidence
Published reports implicating agent	No prior definitive human reports	Limited literature on chromium and pancreatitis
Positive rechallenge	No	Rechallenge not performed for ethical reasons
Exclusion of alternative etiologies	Yes	Normal triglycerides, no alcohol use, normal liver enzymes, normal right upper quadrant ultrasound, MRCP and EUS without obstructive biliary pathology
Objective confirmation of pancreatitis	Yes	Lipase >3000 U/L and imaging-confirmed acute interstitial pancreatitis
Temporal relationship	Yes	Symptom onset 3 d after supplement initiation
Improvement after withdrawal	Yes	Clinical recovery following discontinuation

Animal studies by Solís-Heredia et al provide mechanistic insight into chromium-induced pancreatic injury with dose-dependent hexavalent chromium (Cr[VI]) accumulation within the pancreas and induction of metallothionein, a stress-response protein involved in heavy metal binding.^6^ Pancreatic metallothionein levels exceeded those in the liver and kidney, suggesting organ-specific vulnerability, and were associated with elevated serum α-amylase, indicating exocrine pancreatic injury.^11,12^ Notably, pancreatic exocrine cells appeared more susceptible to chromium toxicity than endocrine islet cells.^6,13^ This distinction is clinically relevant, as our patient presented with elevated lipase and transient hyperglycemia, supporting predominant exocrine involvement. Although the supplement in our case likely contained trivalent chromium (Cr[III]), oxidative stress and redox cycling may generate Cr(VI)-like intermediates in vivo, contributing to inflammation and tissue injury.^12,14–16^

Taken together, these findings support the pancreas as a target organ for chromium toxicity, particularly with high-dose or prolonged supplementation. Identifying the underlying etiology of pancreatitis is crucial, as unrecognized causes such as supplement-induced injury may increase the risk of recurrence and progression to chronic disease. Patra and Das^17^ reported recurrent pancreatitis in 15% of patients, progression to chronic pancreatitis in 13%, and development of diabetes mellitus in 17%, particularly among those with severe or necrotizing disease; 1% developed pancreatic cancer during follow-up. Emerging evidence further supports biological plausibility, with studies demonstrating higher chromium concentrations in pancreatic secretions and an independent 210% increase in the odds of pancreatic cancer associated with elevated chromium levels.^18^ Chromium is known to induce DNA damage and act as a mutagen, raising concerns regarding pancreatic carcinogenesis.^19^

While definitive human data are lacking, this case emphasizes the importance of obtaining a thorough medication and supplement history in patients with idiopathic pancreatitis. Given the widespread and largely unregulated use of nutraceuticals, clinicians should maintain a high index of suspicion for supplement-induced etiologies. Further research is needed to clarify dose-response relationships and the long-term pancreatic effects of chromium exposure in humans.

This case highlights a potential association between chromium supplementation and AP. The importance of thorough history-taking, including the use of over-the-counter and herbal supplements, when evaluating patients with AP, is essential.

## DISCLOSURES

Author contributions: Y. Kagzi: conceptualization, data collection, literature review, manuscript drafting. PL Chintagunta: literature review, manuscript editing. SK Bansal: critical revision of the manuscript for important intellectual content and final approval. IL Balouch: clinical oversight, manuscript review, and final approval.

Y. Kagzi is the article guarantor and accepts full responsibility for the integrity of the work, including the accuracy of the data and analysis.

Financial disclosure: None to report.

Informed consent is obtained for this case report.

## ABBREVIATIONS:

AP: acute pancreatitis
CECT: contrast enhanced computed tomography
Cr (III): trivalent chromium
Cr (IV): hexavalent chromium
CT: computed tomography
DIP: drug-induced pancreatitis
DV: daily value
EUS: endoscopic ultrasound
MRCP: magnetic resonance cholangiopancreaticography
SIP: supplement-induced pancreatitis
